# Differential RNA packaging into small extracellular vesicles by neurons and astrocytes

**DOI:** 10.1186/s12964-021-00757-4

**Published:** 2021-07-10

**Authors:** Xuan Luo, Renée Jean-Toussaint, Ahmet Sacan, Seena K. Ajit

**Affiliations:** 1 Department of Pharmacology and Physiology, Drexel University College of Medicine, 245 North 15th Street, Philadelphia, PA 19102 USA; 2 School of Biomedical Engineering, Science and Health Systems, Drexel University, 3141 Chestnut Street, Philadelphia, PA 19104 USA

**Keywords:** Extracellular vesicles, miRNA signatures, Neurons, Astrocytes, RNA sorting, EXOmotifs

## Abstract

**Background:**

Small extracellular vesicles (sEVs) mediate intercellular communication by transferring RNA, proteins, and lipids to recipient cells. These cargo molecules are selectively loaded into sEVs and mirror the physiological state of the donor cells. Given that sEVs can cross the blood–brain barrier and their composition can change in neurological disorders, the molecular signatures of sEVs in circulation can be potential disease biomarkers. Characterizing the molecular composition of sEVs from different cell types is an important first step in determining which donor cells contribute to the circulating sEVs.

**Methods:**

Cell culture supernatants from primary mouse cortical neurons and astrocytes were used to purify sEVs by differential ultracentrifugation and sEVs were characterized using nanoparticle tracking analysis, transmission electron microscopy and western blot. RNA sequencing was used to determine differential expression and loading patterns of miRNAs in sEVs released by primary neurons and astrocytes. Motif analysis was conducted on enriched miRNAs in sEVs and their respective donor cells.

**Results:**

Sequencing total cellular RNA, and miRNAs from sEVs isolated from culture media of postnatal mouse cortical neurons and astrocytes revealed a distinct profile between sEVs and their corresponding cells. Though the total number of detected miRNAs in astrocytes was greater than neurons, neurons expressed more sEV-associated miRNAs than astrocytes. Only 20.7% of astrocytic miRNAs were loaded into sEVs, while 41.0% of neuronal miRNAs were loaded into sEVs, suggesting differences in the cellular sorting mechanisms. We identified short RNA sequence motifs, or EXOmotifs, on the miRNAs that were differentially loaded or excluded from sEVs. A sequence motif GUAC was enriched in astrocytic sEVs. miRNAs preferably retained in neurons or astrocytes had a similar RNA motif CACACA, suggesting a cell-type-independent mechanism to maintain cellular miRNAs. mRNAs of five RNA-binding proteins associated with passive or active RNA sorting into sEVs were differentially expressed between neurons and astrocytes, one of which, major vault protein was higher in astrocytes than in neurons and detected in astrocytic sEVs.

**Conclusions:**

Our studies suggest differences in RNA sorting into sEVs. These differences in miRNA signatures can be used for determining the cellular sources of sEVs altered in neurological disorders.

**Video abstract**

**Supplementary Information:**

The online version contains supplementary material available at 10.1186/s12964-021-00757-4.

## Background

Extracellular vesicles (EVs) are nanosized membranous particles that are released by cells into the extracellular space and detected in bodily fluids [1]. Subtypes of the EV family, such as exosomes (30–150 nm), microvesicles (100–1000 nm) and apoptotic bodies (100–5000 nm), can be differentiated by their size, morphology, and biogenesis [1, 2]. Small EVs (sEVs) describe vesicles that are smaller than 200 nm according to the guidelines provided by the International Society for Extracellular Vesicles [3]. sEVs primarily contain proteins, RNA and lipids [4, 5] and promote intercellular communication by transferring molecular cargoes from donor cells to neighboring or distal recipient cells [6, 7].

sEVs are stable as their membranous envelope protects their contents from degradation in the extracellular environment [8]. sEV cargo composition is determined by donor cells and their physiological conditions [9]. Thus, specificity of the donor cells or stimuli impacting cellular physiology can affect sEVs composition [4, 10]. Additionally, sEVs may be able to selectively capture cell-specific proteins, RNAs and lipids, contributing to the cargo signature. The mechanisms that control loading of RNAs into extracellular vesicles for secretion remain largely unknown. Studies have shown that unique subsets of RNAs are governed by various loading systems. Non-templated nucleotide additions on the 3’ end of defined miRNA species such as 3’ end adenylation or 3’ end uridylation shifts the relative accumulation of these species in cellular or exosome compartments respectively [11]. Several studies investigating RNA loading into sEVs have focused on RNA-binding proteins (RBPs) [12]. For miRNAs, both a non-selective secretion model [13] and preferential or selective mechanisms of sorting into sEVs have been reported [14–16].

sEVs can cross the blood–brain barrier and deliver cargo with crucial roles in many neurological disorders [17–23]. However, selective isolation of neuron-derived sEVs using peripheral biofluids is currently not feasible because of the lack of a neuron-specific biomarker [24]. Studies have shown that EVs circulating in the peripheral blood are enriched for L1 cell adhesion molecule (L1CAM), a protein found primarily in the nervous system and is used as a neuronal marker [25, 26] and astrocyte-specific glutamine aspartate transporter (GLAST) [27, 28]. Central nervous system (CNS) neuron- and oligodendrocyte-derived exosomes in human peripheral blood have been captured by employing an array of cell-type specific antibodies via surface plasmon resonance imaging [29]. However, it remains difficult to draw definitive conclusions on how brain status can alter the composition of peripheral sEVs cargo.

Information on sEV composition, specifically miRNAs, can be used to predict the cellular source that predominantly contributes to sEVs in circulation. Previous studies of miRNAs in cells and their secreted sEVs have shown significant differences between the populations of miRNAs; certain miRNAs are selectively sorted into sEVs thereby lowering their expression levels in the cells secreting them, while other miRNAs are selectively retained by cells [30, 31]. Proteins and RNAs are sorted in a cell type-specific manner and can provide valuable information as donor cell signatures. We hypothesized that only a subset of cellular miRNAs is sorted into sEVs secreted by neurons and astrocytes, and some of the miRNAs packaged will have cell-specific expression. We isolated sEVs from primary mouse cortical neuronal and astrocytic cultures and refer to them as neuronal sEVs and astrocytic sEVs. RNA sequencing of sEVs and their respective cells was used to elucidate the differences between vesicular and cellular miRNA composition and compare the expression levels of miRNAs between neuronal sEVs and astrocytic sEVs to determine cell-type-specific signatures. Our studies indicate differentially expressed miRNAs encapsulated in sEVs could be used as cell-type-specific signatures for neurons and astrocytes.

## Methods

### Mice

All procedures were conducted in accordance with the NIH Guide for the Care and Use of Laboratory Animals and approved by the Institutional Animal Care & Use Committee of Drexel University College of Medicine. Timed-pregnant CD-1 mice were purchased from Charles River Laboratories (Charles River, NY, USA). All dams were received 13–15 days after impregnation.

### Primary cultures

Primary neuronal and glial cultures were prepared from cortex of pups born the same day or postnatal day one (P0–P1) for neurons, and P2–P4 for astrocytes. In brief, cortices were dissected and incubated at 37 °C for 20 min in Hanks Balanced Salt Solution (HBSS, Corning, VA, USA) containing 10 mM HEPES (Gibco, NY, USA), 7.5 U/mL papain (Worthington Biochemical Corporation, NJ, USA) and 0.1 mg/mL Deoxyribonuclease I (Sigma-Aldrich, MO, USA), followed by gentle trituration with a fire-polished Pasteur pipette. Dissociated neurons were plated in pre-coated T75 flasks or on pre-coated 18-mm coverslips with neurobasal A plating medium (Gibco) containing B-27 Plus (Gibco), 2% (v/v) heat-inactivated fetal bovine serum (FBS, Corning), 2% (v/v) heat-inactivated horse serum (Sigma-Aldrich), GlutaMAX (Gibco) and 50 μg/mL gentamicin (Sigma-Aldrich).

Flasks or coverslips for primary neurons were coated with 0.05% poly-D-lysine (Gibco) (room temperature for 30 min) and 0.05% laminin (Sigma-Aldrich) (37 °C for at least 2 h). Cell plating density was in the range of 800–1000 cells/mm^2^. Plating medium was replaced with 15 mL of neurobasal A maintenance medium supplemented with B-27 Plus, GlutaMAX and 50 μg/mL gentamicin four h to overnight after seeding. Half of the conditioned medium was collected every 24 h and replaced with same volume of fresh warm maintenance medium. On the third day after replacing plating medium, the whole medium was collected. Cells were then washed with phosphate-buffered saline (PBS, Corning), trypsinized with TrypLE Express (Gibco), and pelleted.

For astrocytic cultures, disassociated cells were incubated in Dulbecco’s modified eagle media (DMEM, Corning) containing 10% (v/v) FBS and penicillin–streptomycin (Gibco). After 9–13 days in vitro, flasks were shaken on an orbital shaker (320 rpm) for 6 h to detach microglia and oligodendrocytes. The remaining astrocytes were placed in new flasks for an additional 7–9 days to reach confluency. Then the astrocytes were subcultured again with approximately 160 cells/mm^2^ in uncoated flasks or on uncoated 18-mm coverslips. When confluent, medium was replaced with DMEM containing 10% (v/v) exosome-depleted FBS (#A27208-01, Life Technologies) and penicillin–streptomycin. Conditioned medium and astrocytes were collected 24 h later as mentioned above. All cultures were maintained at 37 °C in a humidified incubator with 5% CO_2_.

### Immunofluorescence

Cells on coverslips were fixed with 4% (w/v) paraformaldehyde (Sigma-Aldrich) for 10 min and permeabilized using 0.1% Triton X-100 (Acros Organics, Belgium) for 15–20 min at room temperature. Non-specific binding sites were blocked by 5% (w/v) normal goat serum (Vector Laboratories, CA, USA) for 45 min. Cells were then incubated with unconjugated mouse anti-MAP2A, 2B (1:500, #MAB378, Millipore, MA, USA) primary antibody overnight at 4 °C. Goat anti-mouse IgG1 Alexa Fluor 546 antibody (1:500, #A-21123, Invitrogen, CA, USA) was used at room temperature for 2 h. Conjugated antibodies, Alexa Fluor 647 conjugated mouse anti-GFAP (1:500, #560298, BD Pharmingen, CA, USA), and FITC-conjugated rat anti-CD11b (1:500, #ab24878, Abcam, MA, USA), were used at room temperature for 2 h.

DAPI (1 μg/mL, Sigma-Aldrich) for nuclear staining was incubated with cells for 10 min at room temperature. Coverslips were then cured in antifade mountant (Invitrogen) overnight at room temperature. Images were acquired with Olympus FluoView FV3000 confocal laser microscope (Olympus, Tokyo, Japan) and processed with integrated software Olympus cellSens platform.

### Isolation of small extracellular vesicles from cell culture supernatants

Small extracellular vesicles (sEVs) were purified from cell culture supernatants of neurons and astrocytes using differential ultracentrifugation at 4 °C as previously described [32, 33] with minor modifications. Briefly, culture supernatants collected were first subject to a centrifugation step of 500 × *g* for 10 min to pellet cell debris, and the supernatants were stored at -80 °C until use. Supernatants were centrifuged at 12,000 × *g* for 30 min (Sorvall RC-5C Plus centrifuge with Sorvall SA-600 fixed-angle rotor, Sorvall Corporate, CT, USA) and passed through a 0.2 µm filter, followed by centrifugation at 110,000 × *g* for 70 min (Optima LE-80 K ultracentrifuge with 50.2 Ti fixed-angle titanium rotor, Beckman Coulter Inc., CA, USA) to pellet sEVs. Pellets were resuspended in PBS and washed at 110,000 × *g* for 60 min (Optima TLX ultracentrifuge with TLA 100.4 rotor, Beckman Coulter, Inc.). sEVs were resuspended in PBS or other solutions specified in each of the following sections. All sEV suspensions were stored at − 80 °C until further use.

### Nanoparticle tracking analysis (NTA)

Size distribution and concentration of sEVs were visualized using NanoSight LM10 equipped with sCMOS camera and 405 nm laser (Malvern Instruments, MA, USA). Briefly, samples in PBS were thawed to room temperature and after a 30 s vortex, diluted to approximately 10^8^ particles/mL with PBS and injected into sample chamber. Five replicates of analysis by 60 s for each diluted sample were captured at 25 °C and processed using NTA 3.3 software.

### Transmission electron microscopy (TEM)

Morphology and size of sEVs were characterized as previously described [34] with minor modifications. All procedures were conducted at room temperature except for embedding. sEVs were re-suspended in 2% (w/v) paraformaldehyde (Electron Microscopy Sciences) in 0.1 M Sorensen's phosphate buffer (0.1 MPB, Electron microscopy sciences). Ten μl of suspension was placed on each formvar-coated electron-microscopy grid for 10 min. Copper grids (Electron Microscopy Sciences) were used for negative staining. Alternatively, samples for immuno-labeling were absorbed on nickel grids (Electron Microscopy Sciences) and blocked by 5% (w/v) bovine serum albumin (BSA, AMRESCO, OH, USA) for 10 min, followed by incubation with mouse CD81 (1:50, #sc-166029, Santa Cruz Biotechnology, TX, USA) for 30 min and 10 nm gold-conjugated donkey anti-mouse IgG secondary antibody (1: 20, #25815, Electron Microscopy Sciences) for 20 min. Negative-staining or immunolabeling samples were fixed on the grids using 1% glutaraldehyde (Sigma-Aldrich) for 5 min, contrasted on 1% uranyl acetate (Electron Microscopy Sciences) for 2 min. Samples were then embedded in 0.2% uranyl acetate in 0.16% (w/v) methyl cellulose (Sigma-Aldrich) for 10 min on ice. Grids were imaged on a JEM-1230 transmission electron microscope (JEOL USA, MA, USA) at 80 kV with 100,000 × magnification.

### Western blotting

Cells and sEVs from primary neuronal and astrocytic cultures were re-suspended in RIPA buffer (Sigma-Aldrich) containing protease inhibitors (Thermo Fisher Scientific, MA, USA). Protein concentrations were measured using DC protein assay (Bio-Rad, CA, USA) or Micro BCA Protein Assay kit (Thermo Scientific) according to the manufacturer’s instructions. Equal amounts of protein lysates were resolved on 10% SDS-PAGE gel, transferred to PVDF membrane, and blocked in Odyssey TBS blocking buffer (LICOR Biosciences, NE, USA) or 5% non-fat dry milk at room temperature for 1 h. Membranes were incubated with primary antibodies in Tris-Buffered Saline with 0.1% TWEEN 20 (TBST) containing 10% (v/v) blocking buffer overnight at 4 °C, then incubated with secondary antibodies in TBST at room temperature for 1 h. Prior to imaging, membranes probed with HRP antibodies were incubated in SuperSignal West Femto Substrate (Thermo Scientific) for 5 min at room temperature. Total protein loading was determined using SYPRO Ruby protein gel stain (Bio-Rad) following manufacturer’s instructions. Images were acquired using Odyssey Fc imaging system (LI-COR Biosciences) or FluorChem M system (ProteinSimple, CA, USA). Primary antibodies used were as following: rabbit calnexin (1:500, # ab10286, Abcam), mouse anti-CD81 (1:500, #sc-166029, Santa Cruz Biotechnology), rabbit anti-Hsp70 (1:2000, #ab94368, Abcam), CD63 (1:200, #216130, Abcam), major vault protein (1:500, #16478-1-AP, Proteintech), annexin A2 (1:500, #sc28385, Santa Cruz Biotechnology), beta actin (1:500, #ab8227, Abcam). Secondary antibodies used were, HRP-conjugated goat anti-mouse IgG (1:5000, #ab6789, Abcam), donkey anti-rabbit IgG (1:5000, #ab16284, Abcam), 680RD donkey anti-mouse IgG (#926-68072, LI-COR Biosciences), and 800CW donkey anti-rabbit IgG (#926-32213, Li-COR Biosciences).

### RNA isolation from cells and small extracellular vesicles

RNA was isolated using the miRVana kit (Life technologies). Total RNA was isolated from cells and sEVs immediately after the purification of sEVs, according to the manufacturer’s instructions with the incorporation of RNAse-free DNAse (Epicentre, WI, USA) treatment [33]. RNA samples obtained from 3 independent experiments for neurons, astrocytes, and their sEVs, were stored at − 80 °C until shipment to Beijing Genomics Institute for sequencing. RNA concentration, RIN value, 28S/18S ratio and fragment length distribution for all samples were validated before sequencing using Agilent RNA 6000 nano chip on an Agilent 2100 bioanalyzer (Agilent, CA, USA).

### Library construction and RNA sequencing

Enriched mRNA from total RNA via oligo dT selection or rRNA depletion was reverse transcribed, end-repaired, 3’ adenylated as well as adaptor ligated, and purified. Purified cDNA was amplified, denatured, and circularized into single-stranded circular DNA (ssDNA circle) [35]. Small RNA fragments including mature miRNAs and other regulatory small RNA molecules were purified from total RNA by separating the 18–30 nucleotide stripe from a 15% urea-PAGE gel. miRNA fragments were 5’ adenylated, adaptor ligated and reverse transcribed. The resulting cDNA was amplified, purified from 100–120 base-pair fragments, and circularized into ssDNA circle [36]. DNA nanoballs generated from ssDNA circle were sequenced as 50 bp single reads on the BGISEQ-500 platform with patterned nanoarrays, generating an average of 30 million reads per sample.

### Statistical and bioinformatics analysis

After sequencing, low quality and adaptor sequences (3.1% of total reads for mRNAs and 35.61% for miRNAs) were filtered out to obtain clean reads. Clean reads were mapped against the reference genome (Rfam12_ncRNA) using Bowtie2 [37]. Expression levels were calculated using fragments per kilobase million (FPKM) for mRNA and transcripts per kilobase million (TPM) for miRNA. For mRNA data, differentially expressed genes (DEGs) between neuronal and astrocytic cells were determined using DESeq2 based on a negative binomial distribution [38]. DEGs were identified with an absolute fold change ≥ 2 and a positive false discovery rate ≤ 0.05. Enriched Gene Ontology (GO) analyses for mRNA derived from neurons and astrocytes were performed and summarized using the Database for Annotation, Visualization and Integrated Discovery (DAVID) Bioinformatics Resources 6.7 [39, 40] and REVIGO [41], respectively. For miRNA data, a presence-absence analysis as well as differential expression analysis were performed. The presence of a miRNA in a group of samples was defined if the TPM of that miRNA was larger than 0.1 for all samples of the group [42]. Differentially expressed miRNAs were determined with an absolute fold change ≥ 2 (calculated from the geometric means of TPM values) and *p*-value ≤ 0.01 from a two-sample t-test of the log2-transformed TPM values.

Purity of cell cultures and protein expression studies were performed in triplicate. Data are represented as mean ± standard deviation. The purity of primary cultures was determined by the presence of MAP2A^+^ cells for neurons or GFAP^+^ for astrocytes in the total number of cells stained with DAPI. Statistical analysis was performed using Prism 8.2.1. Protein expressions between cell types were compared using a two-tailed unpaired t-test. *P*-value < 0.05 was considered statistically significant.

### Motif analysis

Analysis of over-represented motifs was conducted on enriched miRNAs in sEVs and their respective donor cells using the MEME Suite [43]. A zero or one site per sequence (ZOOPS) model was used to identify motif site distribution on miRNAs. All mouse miRNAs annotated in TargetScan [44] were used as background with a zero-order Markov model to normalize for biased nucleotide distribution.

## Results

### Purity of primary neurons and astrocytes

**Fig. 1:**
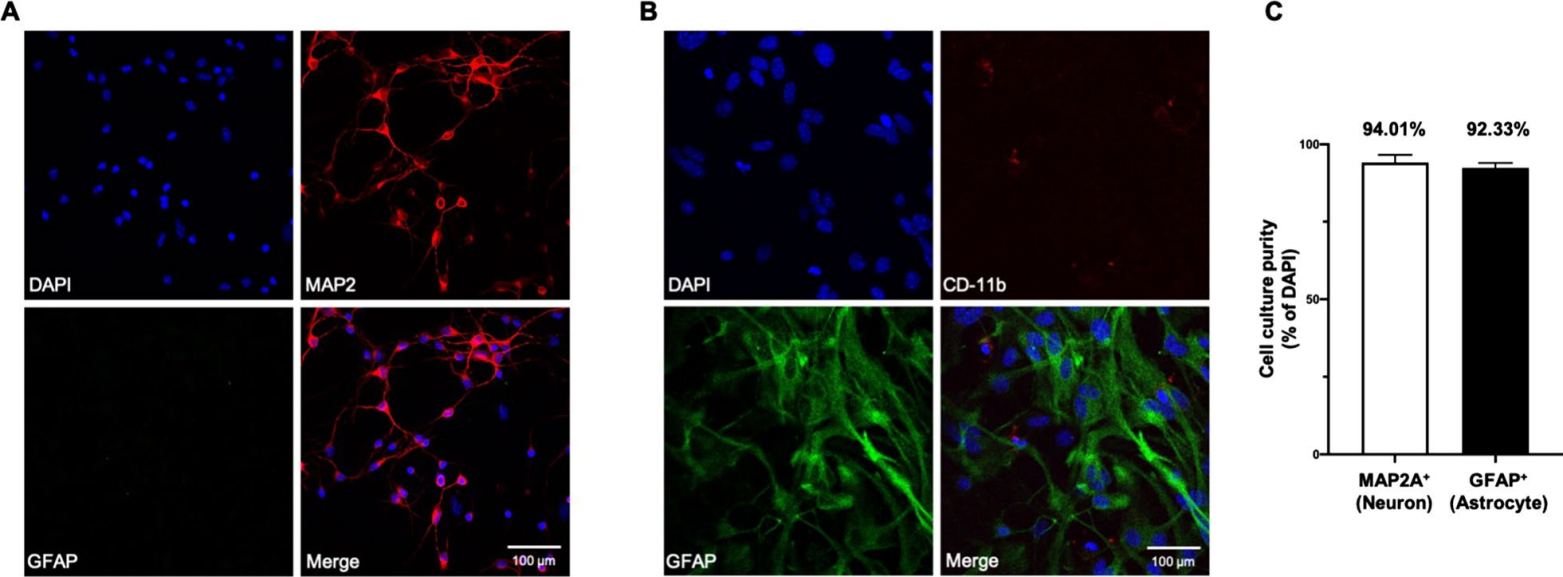
Purity of neuronal and astrocytic cultures. **a** The purity of the neuronal culture was confirmed using neuronal marker MAP2 (microtubule associated protein 2) (red) and absence of astrocytic marker GFAP (glial fibrillary acidic protein) (green). Most cells with nuclei marker DAPI (blue) were also stained with MAP2, while few green GFAP labeled astrocytes were observed. **b** The astrocytic culture was enriched in GFAP (green) while the microglial marker CD-11b (red) was almost completely absent, indicating purity of DAPI-stained astrocytes. Scale bars = 100 μm. **c** Quantification of percentage purity of neuronal cultures (MAP2A^+^ cells, left) and astrocytic cultures (GFAP^+^ cells, right) in the total number of cells stained with DAPI. The data represents three independent experiments and are presented as mean ± standard deviation

Before isolating cell specific sEVs, we confirmed the purity of primary mouse cortical neuronal or astrocytic cultures using immunofluorescence (Fig. 1) with cell-specific markers. We stained cultures for the neuronal marker microtubule associated protein 2 (MAP2) and the astrocyte marker glial fibrillary acidic protein (GFAP). To further confirm culture purity, we stained neuronal cultures for GFAP and stained astrocytic cultures for the microglial marker CD-11b. Both cultures were enriched in their specific markers, as shown by confocal microscopy (Fig. 1). The neuronal culture had very few astrocytes, and similarly, few microglia were observed in the astrocytic culture, confirming more than 90% purity for cultured neurons and astrocytes (Fig. 1c).

### Characterization of small extracellular vesicles (sEVs) isolated from cell culture supernatant

**Fig. 2:**
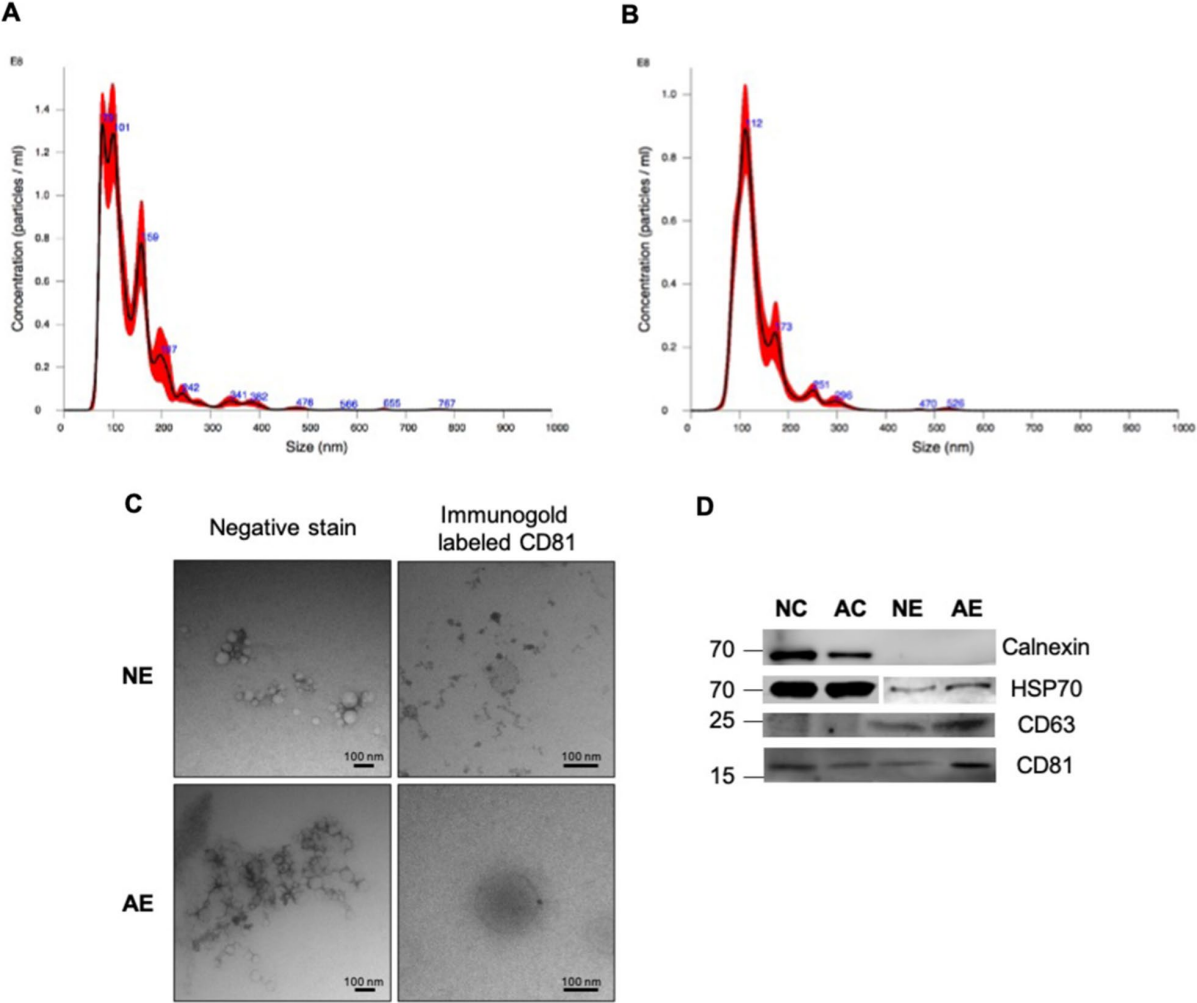
Characterization of sEVs derived from primary neurons and astrocytes. **a** Nanoparticle tracking analysis (NTA) of neuronal sEVs showing a mean diameter of 137.2 ± 2.7 nm and a concentration of 1.07 × 10^10^ ± 8.51 × 10^8^ particles/mL. **b** NTA showing astrocytic sEVs with an average diameter of 135.6 ± 2.2 nm and concentration of 5.68 × 10^9^ ± 2.71 × 10^8^ particles/mL. **c** Transmission electron microscopy (TEM) confirmed that most sEV diameters were to be less than 150 nm and immunogold labeling showed the presence of CD81. Scale bars = 100 nm. **d** Western blot analysis of three independent experiments showed that sEVs were enriched in EV markers HSP70, CD63 and CD81. Calnexin, an endoplasmic reticulum/Golgi marker, was present only in cell lyases but absent in sEV preparations, confirming the purity of sEVs. Ten μg of total protein was loaded per lane. NC: neurons, AC: astrocytes, NE: sEVs from neurons, AE: sEVs from astrocytes

sEVs derived from the media of neuronal and astrocytic cultures were characterized for size using nanoparticle tracking analysis (NTA). Neuronal sEVs had a mean diameter of 137.2 ± 2.7 nm (Fig. 2a) and a concentration of 1.07 × 10^10^ ± 8.51 × 10^8^ particles/mL while the concentration of astrocytic sEVs was 5.68 × 10^9^ ± 2.71 × 10^8^ particles/mL with a mean diameter of 135.6 ± 2.2 nm (Fig. 2b). Transmission electron microscopy (TEM) confirmed that the majority of sEVs had a diameter less than 150 nm, and immunogold labeling showed the presence of EV protein tetraspanin CD81 on the surface (Fig. 2c). Western blot analysis showed that the isolated sEVs were enriched in EV markers HSP70, CD63, in addition to CD81 (Fig. 2d). We probed for contaminants of EV preparations using calnexin, an endoplasmic reticulum/Golgi marker. Calnexin was only present in cell lysates but absent in sEV preparations (Fig. 2d) confirming the purity of sEVs preparations.

### RNA sequencing and analysis

**Fig. 3:**
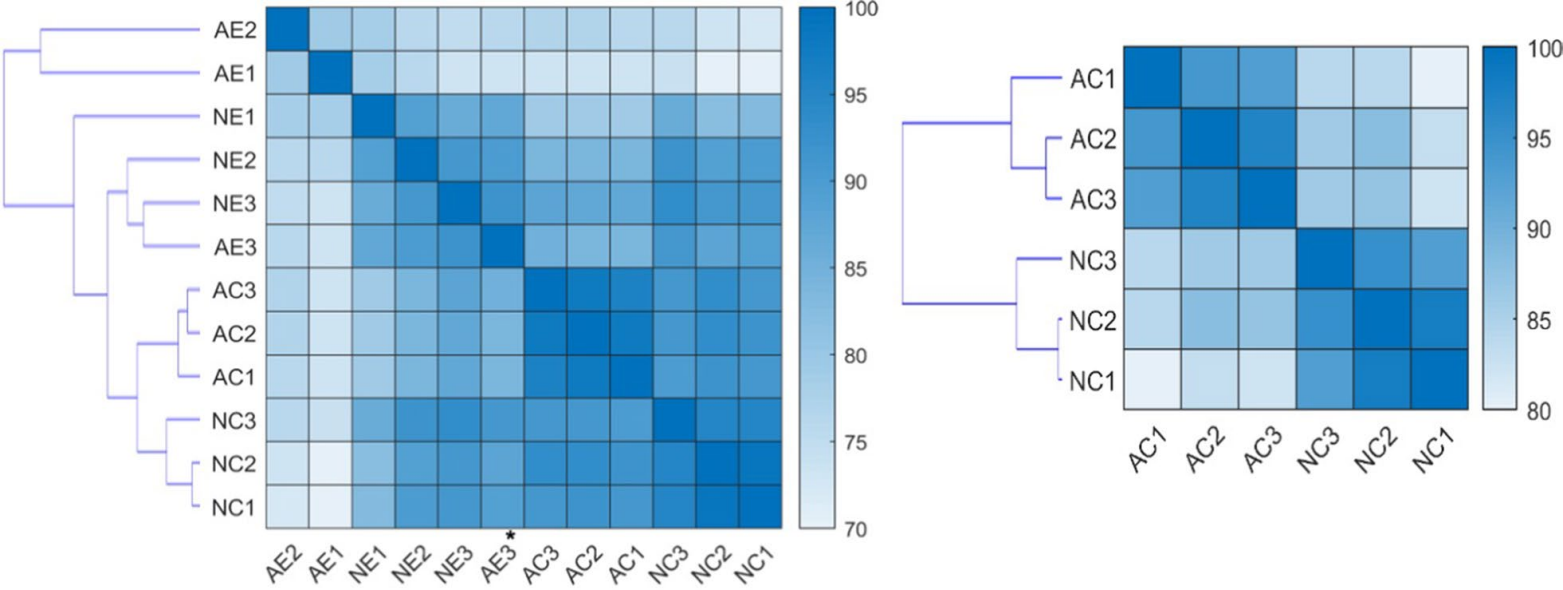
Heatmap and hierarchical clustering of pairwise correlations of samples. (Left) Cellular and sEV miRNA data. (Right) Cellular mRNA data. NC: neurons, AC: astrocytes, NE: sEVs from neurons, AE: sEVs from astrocytes. Color scales represent Pearson correlation values between pairs of samples, shown as percentages. Asterisk indicates that astrocytic sEV sample (AE3) is more similar to neuronal sEVs than other astrocytic sEV samples (AE2 and AE1)

We sequenced total RNA from cellular and sEV samples, where each group had three biological replicates. Before sequencing, we confirmed the integrity and quality of RNA derived from neurons, astrocytes, and their respective sEVs. As previously reported, cellular RNA samples had prominent 18S and 28S ribosomal RNA peaks, which were not observed in sEV RNA samples [45]. The sEV samples had peaks around 25–200 nucleotides, indicating the presence of small RNAs (Additional file 1: Figure S2). While we conducted small RNA sequencing for total RNA from cells and sEVs, we only performed transcriptome sequencing for cellular RNA. We normalized miRNA expression to transcripts per kilobase million (TPM) and calculated gene expression levels using fragments per kilobase million (FPKM). We performed correlation analysis and hierarchical clustering using the Pearson correlation coefficients between pairs of samples from both analyses, showing that the samples from the same cell type and from cellular versus sEV origin had higher correlations and were clustered together (Fig. 3). The exception to this was one of the astrocytic sEV samples (AE3, shown with an asterisk in Fig. 3), which was more similar to the neuronal sEVs than to the other astrocytic sEVs. Note that the correlation analysis and clustering were done using the overall mRNA and miRNA expression levels.

### Selective incorporation of miRNA in sEVs

**Fig. 4:**
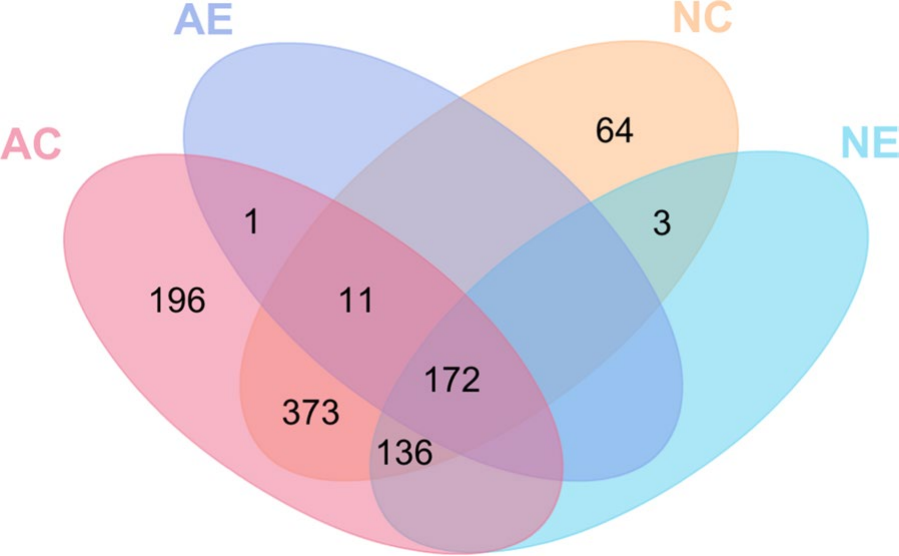
Number of unique and overlapping miRNAs in neurons, astrocytes, and their respective sEVs. miRNA profiles in neurons and astrocytes are distinct in spite of considerable overlap with a total of 67 miRNAs observed only in neuronal sEVs and neurons, while a total of 197 miRNAs detected only in astrocytic sEVs and astrocytes. NC: neurons, AC: astrocytes, NE: sEVs from neurons, AE: sEVs from astrocytes

Studies have shown that donor cells selectively incorporate only a certain subset of intracellular miRNAs into sEVs, and the remaining miRNAs are retained within the cells [46]. We hypothesized that this phenomenon would also occur in postnatal cortical cells and their corresponding secreted sEVs. The presence-absence analysis showed that 759 miRNAs were present in neurons, and 889 miRNAs in astrocytes (Fig. 4 and Additional file 2: Table S1). Sixty-seven miRNAs were exclusively present in neurons while 197 miRNAs were exclusive to astrocytes, indicating that miRNA profiles in neurons and astrocytes are distinct, despite considerable overlap.

As expected, cells had more miRNAs than nanosized sEVs, and all the miRNAs present in sEVs were also found in the donor cells, confirming that only a subset of the cellular miRNAs can be incorporated into sEVs. Although more miRNAs were detected in astrocytes than in neurons, neurons expressed more sEV-associated miRNAs compared to astrocytes. To be specific, only 20.7% of the cellular astrocytic miRNAs were loaded into sEVs, while 41.0% of the cellular neuronal miRNAs were loaded into sEVs, suggesting differences in the cellular sorting mechanisms. Interestingly, neuronal sEVs lacked 11 miRNAs expressed in neurons, astrocytes, and astrocytic sEVs (Additional file 2: Table S1), implying a specific regulatory mechanism for packaging these miRNAs into astrocytic sEVs instead of neuronal sEVs. Similarly, astrocytic sEVs lacked 136 miRNAs expressed in neurons, astrocytes and neuronal sEVs (Additional file 2: Table S1), again suggesting the involvement of selective incorporation of miRNAs in a cell-specific manner. In addition, three miRNAs (mmu-miR-7a-2-3p, mmu-miR-135a-1-3p, and mmu-miR-144-3p) were only found in neurons and neuronal sEVs while mmu-miR-5119 was only expressed in astrocytes and their sEVs.

We then explored the differential expression of miRNAs between sEVs and their respective donor cells (Additional file 3: Table S2). There were 14 specifically enriched as well as 75 specifically diminished miRNAs in neuronal sEVs compared to total neuronal RNAs (Table 1 and Additional file 3: Table S2). Similarly, nine miRNAs were significantly enriched in astrocytic sEVs while 168 miRNAs were specifically retained in astrocytes (Table 2 and Additional file 3: Table S2). These two observations suggest a selective sorting of miRNAs into sEVs that is more profound in astrocytes than in neurons. Notably, mmu-miR-122-5p, mmu-miR-126a-3p and mmu-miR-126a-5p were both enriched in sEVs from neurons and astrocytes, suggesting a common sorting mechanism for these three miRNAs. Together, the distinct miRNA profiles between sEVs and their respective cells suggest the sorting of miRNAs into sEVs is precisely regulated and could be specific to cell types.

**Table 1:** Differentially expressed miRNAs in neuronal sEVs compared to neurons

miRNA	Fold change (NE/NC)	*p*-value
mmu-miR-122-5p	335.6	1.6E−3
mmu-miR-122-3p	36.4	2.2E−3
mmu-miR-126a-3p	26.6	8.2E−4
mmu-miR-709	24.7	4.0E−3
mmu-miR-143-3p	24.2	4.1E−6
mmu-miR-365-3p	22.1	1.9E−3
mmu-miR-145a-3p	20.3	2.1E−3
mmu-miR-199a-3p	14.7	7.0E−4
mmu-miR-145a-5p	14.4	3.7E−6
mmu-miR-126a-5p	11.2	2.3E−3
mmu-miR-10a-5p	10.7	9.3E−3
mmu-miR-224-5p	4.6	2.8E−3
mmu-miR-30d-5p	2.4	7.0E−3
mmu-miR-135a-1-3p	2.1	9.5E−3

NC, neurons; NE, sEVs from neurons

**Table 2:** Differentially expressed miRNAs in astrocytic sEVs compared to astrocytes

miRNA	Fold change (AE/AC)	*p*-value
mmu-miR-150-5p	730.2	1.4E−3
mmu-miR-122-5p	598.2	1.4E−4
mmu-miR-466i-5p	296.5	1.0E−3
mmu-miR-486a-5p	167.0	3.4E−3
mmu-miR-126a-3p	112.4	2.4E−3
mmu-miR-126a-5p	28.9	8.2E−3
mmu-miR-486b-3p	15.3	2.2E−4
mmu-let-7a-1-3p	3.9	3.4E−3
mmu-miR-25-3p	2.7	7.2E−3

AC, astrocytes; AE, sEVs from astrocytes

We next hypothesized that sEVs secreted by neurons and astrocytes may also have distinct miRNA signatures that are unique to the donor cells. Thus, we performed differential expression analysis for miRNAs derived from neuronal sEVs and astrocytic sEVs. We observed differential expression of eight miRNAs, six of which had higher expression in neuronal sEVs compared to astrocytic sEVs (Table 3).

**Table 3:** Cell-type-specific miRNA signatures in neuronal and astrocytic sEVs

miRNA	Fold change (NE/AE)	*p*-value
mmu-miR-5100	39.7	2.1E−3
mmu-miR-135a-2-3p	26.8	1.3E−3
mmu-miR-429-3p	26.7	6.8E−3
mmu-miR-669l-5p	23.5	4.8E−4
mmu-miR-374b-5p	15.7	2.9E−3
mmu-miR-450a-5p	5.6	9.7E−3
mmu-miR-23a-3p	− 2.5	9.8E−3
mmu-miR-146a-5p	− 3.2	8.7E−3

NE, sEVs from neurons; AE, sEVs from astrocytes

### Short motifs could be a possible mechanism for shuffling and retaining cellular miRNAs

**Fig. 5:**
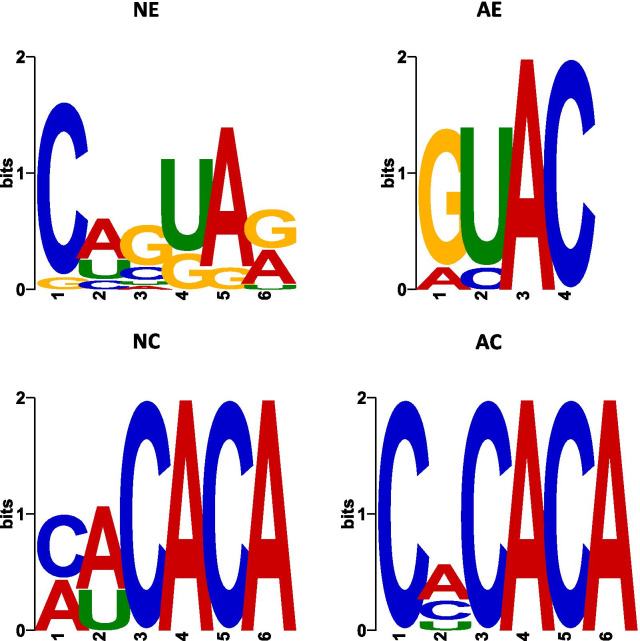
Over-represented motifs on miRNAs differentially expressed in neurons, astrocytes, and their respective sEVs. Sequence motif patterns of 4- to 8-nucleotides on differentially expressed miRNAs in sEVs and their corresponding cells. One motif was identified for each set of specifically upregulated miRNAs (also see Table 4, and Additional file 4: Table S3). NC: neurons, AC: astrocytes, NE: sEVs from neurons, AE: sEVs from astrocytes

We examined sequence motif patterns of 4- to 8-nucleotides on differentially expressed miRNAs in secreted sEVs and their corresponding cells using the MEME Suite [43]. One motif was identified for each set of specifically upregulated miRNAs (Fig. 5, Table 4, and Additional file 4: Table S3). Specifically, five out of nine miRNAs that were enriched in astrocytic sEVs had a GUAC motif (E-value = 3.7), while nine of 14 differentially upregulated miRNAs in neuronal sEVs shared a CWGUAR motif. Highly represented miRNAs in neurons or astrocytes showed a similar CACACA motif (E-value = 40 in neurons and E-value = 2.6 in astrocytes), including 11 out of 161 miRNAs in astrocytes and nine out of 73 in neurons.

**Table 4:** RNA sequence motifs over-represented in miRNAs upregulated in neurons, astrocytes, and their respective sEVs

Group	Motif	# of miRNAs having the motif	Total # of upregulated miRNAs	E-value
NE	CWGUAR	9	14	7.1
AE	GUAC	5	9	3.7
NC	MWCACA	9	73	40.0
AC	CMCACA	11	161	2.6

NC, neurons; AC, astrocytes; NE, sEVs from neurons; AE, sEVs from astrocytes

### Differential expression of RNA-binding proteins

**Fig. 6:**
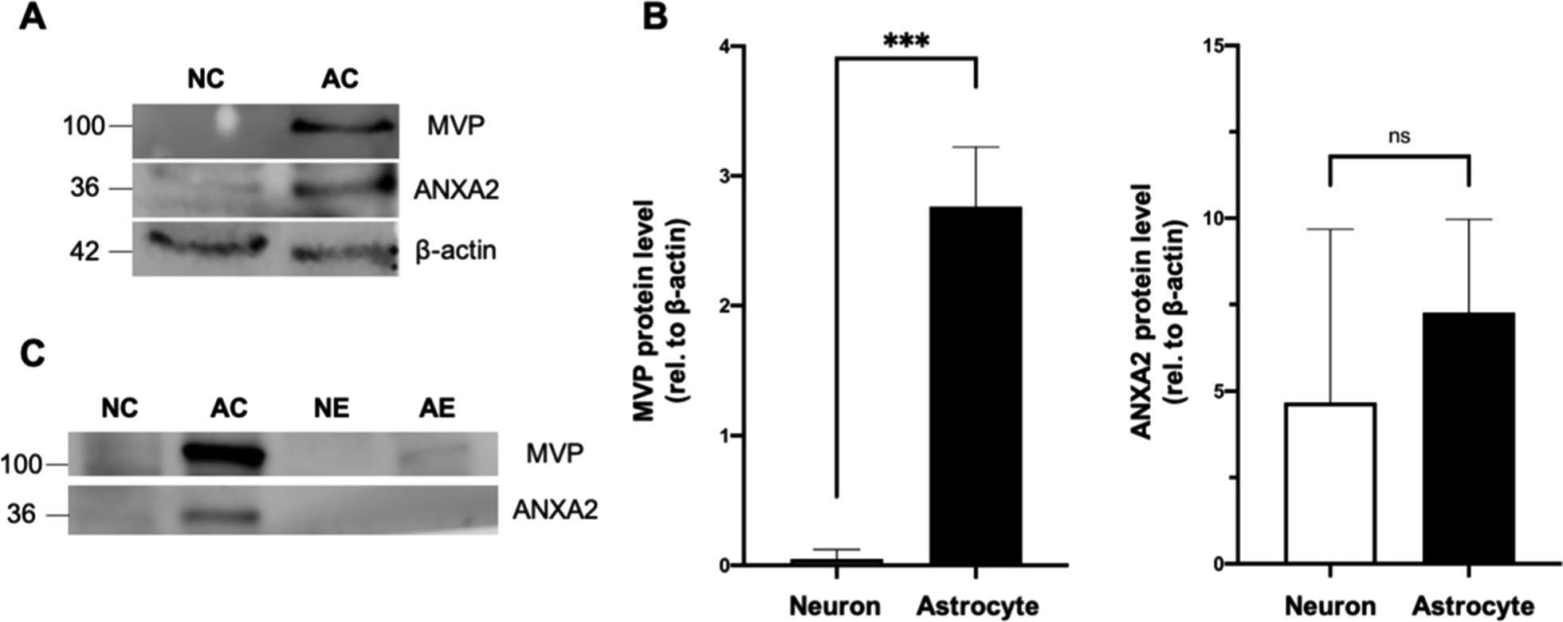
Western blot analysis of major vault protein (MVP) and annexin A2 (ANXA2) in primary cortical neurons and astrocytes. **a** Representative images of the protein expression of MVP, ANXA2, and loading control β-actin in neurons and astrocytes. **b** Quantification of MVP and ANXA2 expression (normalized to the expression of β-actin) is shown as mean ± standard deviation. n = 3 independent experiments. ***p-value < 0.0005; ns: not significant; two-tailed unpaired t-test. Twenty μg of total protein was loaded per lane. **c** Representative images showing the expression of MVP and ANXA2 in primary cortical neurons, astrocytes, and their sEVs (n = 2). Ten μg of total protein was loaded per lane. NC: neurons, AC: astrocytes, NE: sEVs from neurons, AE: sEVs from astrocytes

Our RNA-seq of neuronal and astrocytic cells showed that 2446 and 2438 mRNAs were specifically enriched in neurons and astrocytes, respectively (Additional file 5: Table S4). Gene Ontology (GO) analysis performed on differentially expressed genes in neurons and astrocytes was summarized (Additional file 1: Figure S3), and as expected, biological processes for upregulated genes in neurons were enriched in nerve impulse and neurotransmitter processes while enriched genes in astrocytes were related to tube development and extracellular matrix organization. Among the differentially expressed genes, we specifically focused on RNA-binding proteins (RBPs) because of their known role as possible mediators for loading RNA into sEVs [46]. RBPs can form ribonucleoprotein complexes with single- or double-stranded RNA through specific interactions. In a previous report, 30 RBPs were identified in exosomes in complex with different RNA species in a cell-free assay, 20 of which were associated with exosomal RNAs [46]. This indicates that exosomal RNAs exist predominantly as RNA-binding protein complexes. Therefore, we compared the mRNA expression of these RBPs in neurons and astrocytes. We observed that three RBPs, heterogeneous nuclear ribonucleoprotein H1 (hnRNPH1), argonaute 1 (AGO1) and argonaute 4 (AGO4), had higher mRNA expression in neurons, while annexin A2 (ANXA2) as well as major vault protein (MVP), were expressed at significantly lower levels compared to astrocytes (Table 5 and Additional file 5: Table S4). We further examined the protein levels of MVP and ANXA2 in neurons and astrocytes (Fig. 6). MVP demonstrated higher expression in astrocytes compared to neurons (*p* < 0.0005; two-tailed unpaired t-test), which is in line with the mRNA expression. However, there was no statistically significant difference in ANXA2 protein between neurons and astrocytes (Fig. 6b). In addition, MVP was present in astrocytic sEVs but absent in neuronal sEVs (Fig. 6c).

**Table 5:** Differential expression of mRNA encoded for RNA-binding proteins (RBPs) in neurons compared to astrocytes

mRNA	Up/down (NC/AC)	Protein name	Reported sorting mechanism
*Hnrnph1*	Up	Heterogeneous nuclear ribonucleoprotein H1	Passive [46]
*Ago1*	Up	Argonaute 1	–
*Ago4*	Up	Argonaute 4	–
*Mvp*	Down	Major vault protein	Passive [46]/active [71]
*Anxa2*	Down	Annexin A2	Passive [72]

NC, neurons; AC, astrocytes; Up, upregulated; down, downregulated

## Discussion

Identification of cellular origin of the sEVs in the circulation, specifically from CNS cells such as neurons, has been challenging due to the absence of specific protein markers [24]. The two-way communication between astrocytes and neurons contributes to the maintenance of brain homeostasis [47]. Astrocytes, a crucial component of the blood brain barrier (BBB), protect neurons by maintaining the highly regulated CNS internal milieu. Malfunction of the BBB in neurodegenerative diseases contributes to neuronal damage, synaptic dysfunction and loss of neuronal connectivity [48]. sEVs can cross the BBB, and EVs released by both types of cells could be present in circulation [49]. Thus, identifying miRNAs that are exclusively expressed in sEVs from distinct donor cells may enable us to define the cellular origin of the circulating sEVs [50, 51]. Here, we investigated miRNA signatures from postnatal mouse cortical neuronal and astrocytic cultures and their sEVs to better understand which of the cellular miRNAs were packed into sEVs and to identify unique miRNAs in sEVs released from the two different cell types.

We observed both a unique and overlapping presence of miRNAs in neurons, astrocytes, and their respective sEVs. The number of miRNAs present in astrocytes were higher than neurons, with astrocytes expressing 889 miRNAs and neurons expressing 759 miRNAs. sEV-associated miRNAs were also different in neuronal sEVs (311 miRNAs) and astrocytic sEVs (184 miRNAs). A previous study detected a total of 131 distinct miRNAs in EVs secreted by primary rat cortical astrocyte cell cultures [52]. The difference in number could be attributed to the method used for miRNA profiling and species used. We used RNA sequencing for mouse sEVs to theoretically detect any miRNA species while the previous study used nCounter Rat miRNA expression assay (Nanostring technologies) that is designed to specifically probe a total of 423 rat miRNAs [52]. Another study on mouse astrocytes has used qPCR with TaqMan rodent miRNA arrays to probe 752 mature miRNA species and identified 168 of these miRNAs present in exosomes, with 54 of these miRNAs enriched in exosomes compared to astrocytes [53]. Only three of these miRNAs enriched in exosomes, mir-25, miR-126-5p (or mmu-miR-126a-5p), and mir-150, matched the miRNAs that we identified to be differentially enriched in astrocytic sEVs. In a previous study, miR-135a was specifically enriched in astrocytes as compared to neurons and oligodendrocytes isolated from neonatal rats [54]; miR-135a was also detected in embryonic mouse hippocampal neurons [55]. In our study, we observed miR-135a (mmu-miR-135a-1-3p) to be present only in neurons and their sEVs but was absent in astrocytes and their sEVs. The differences in our results may be attributed to the species, the region of the brain the sEV donor cells were obtained, cell culture growth conditions and the RNA quantification methods.

All miRNAs expressed in sEVs overlapped with those identified in their corresponding donor cells. This confirmed that only a subset of the cellular miRNAs can be incorporated into sEVs. Only 20.7% of cytosolic miRNAs in astrocytes were loaded into astrocytic sEVs, while 41.0% of neuronal miRNAs were loaded in neuronal sEVs. Despite the distinct sEV-associated miRNA profiles, the different percentage of cellular miRNAs sorted by neurons and astrocytes into respective sEVs implies that the sorting machineries work differently in these cells, and the selectivity of miRNAs packaged into sEVs may be more profound in astrocytes than neurons. For example, 11 miRNAs detected in neurons and astrocytes were expressed in astrocytic sEVs, but 136 miRNAs in both cells were exclusively expressed in neuronal sEVs but not astrocytic sEVs. Collectively, this demonstrates a tight regulation of sorting miRNAs into sEVs in a cell-type-specific manner. Regardless of the overlapping miRNAs in both cell types, 139 and 12 miRNAs were unique in neuronal and astrocytic sEVs respectively. Neuronal sEVs exclusively expressed three miRNAs (mmu-miR-7a-2-3p, mmu-miR-135a-1-3p, mmu-miR-144-3p), and astrocytic sEVs exclusively expressed one miRNA (mmu-miR-5119). mmu-miR-7a-2-3p [56] and mmu-miR-144-3p [57] were shown to be downregulated in primary neurons after oxygen–glucose deprivation (OGD), but overexpression of these two miRNAs resolved the hypoxia–ischemia-induced neuronal apoptosis. miR-135a expressed in mouse hippocampal neurons was reported to be essential for the regulation of excitatory neurotransmission [54, 55]. The presence of these miRNAs may help determine the cellular sources of the sEV-encapsulated miRNAs in circulation. Of note, these cell-type-specific sEV miRNAs were also reported to be present in a variety of cells. miR-7a is also expressed in sensory neurons of dorsal root ganglion and plays an important role in the maintenance of neuropathic pain [58]. mmu-miR-5119 implicated in enhanced anti-tumor cell immune response, was also enriched in microglia in developing brain and spleen dendritic cells [59, 60]. Therefore, additional studies are needed to assess if other cell types could contribute these miRNAs in circulation.

Our quantitative demonstration that astrocytes tend to exclude miRNAs from sEVs suggests they have a more stringent sorting of miRNAs into sEVs. However, mmu-miR-122-5p, mmu-miR-126a-3p and mmu-miR-126a-5p were highly enriched in both types of sEVs, suggesting that neurons and astrocytes may also share common mechanisms for loading these three miRNAs into sEVs. Downregulation of miR-122 was observed in mouse neuroblastoma cells after OGD, and inversely, addition of miR-122 resulted in the resolution of ischemic neuronal death [61]. miR-126 which is abundant in mouse brain endothelial cells, is also expressed in astrocyte-derived exosomes to a lesser extent [62]. Several RNA-binding proteins have been identified to be associated with selective packaging of miR-122, including lupus La protein [63] and human ELAV protein HuR under stress, both verified in human cell lines [64]. The gene expression of these two proteins (*Ssb* for La protein and *Elavl1* for HuR) were not significantly different between neurons and astrocytes (Additional file 5: Table S4), suggesting the highly regulated sorting of mmu-miR-122-5p, mmu-miR-126a-3p and mmu-miR-126a-5p could be attributed to La protein and HuR. Further investigation will help us understand how neurons and astrocytes select cellular miRNAs into their sEVs and what sorting mechanisms are unique to neurons and astrocytes.

We also identified six neuron-specific sEV miRNA signatures and two sEV miRNAs unique to astrocytic sEVs (Table 3). miR-23a-3p, miR-126-3p, miR-146a-5p, and miR-5100 were identified as some of the most abundant miRNAs in the temporal lobe neocortex of human brain [62]. Although the role of miR-5100 remains elusive, it has been identified as a disease biomarker for cancer [65] and lupus [66]. miR-23 is involved in neuronal specification, and miR-146a implicated in the prevention of neuroligin 1-dependent synaptogenesis, and both were enriched in astrocytes compared to neurons [54, 67]. Our results are in line with these observations (Additional file 3: Table S2). Interestingly, mmu-miR-23a-3p and mmu-miR-146a-5p, the two miRNAs enriched in astrocytes, were also highly expressed in in astrocytic sEVs compared to neuronal sEVs. We and others have observed miR-146a to have an anti-inflammatory role [68]. Yet, the uptake of these sEVs in recipient cells and the contribution of these sEV-encapsulated miRNAs in gene regulation need to be further elucidated.

RNA packaging into sEVs during biogenesis may be controlled by RBPs that form complexes with RNA. We investigated the expression levels of RBPs in neurons and astrocytes. A previous report showed that 23 of 30 RBPs discovered in exosomes were in a complex with cellular miRNAs and/or exosomal RNAs [46]. We examined the mRNAs of all 30 identified RBPs, and most of them were not significantly differentially expressed between neurons and astrocytes. However, the mRNAs for major vault protein (MVP) and annexin A2 (ANXA2) were upregulated in astrocytes compared to neurons, while the mRNA levels for *Hnrnph1*, argonaute 1 (*Ago1*) and argonaute 4 (*Ago4*) were lower in astrocytes. A recent study that characterized sEV cargo proteins derived from human primary astrocytes showed that MVP was present in two of the five control sEVs and in all the same samples after the cells were treated with IL-1β [69]. ANXA2 was detected in all the samples. For the downregulated genes, hnRNPH1 protein was detected only in one control sample and both AGO proteins were absent [69]. Furthermore, proteomics analysis of cell preparations from mouse brain showed that MVP and ANXA2 were upregulated, whereas hnRNPH1, AGO1 and AGO4 were downregulated in astrocytes compared to neurons [70]. These results are in agreement with our observations of the same RBP mRNA levels.

MVP, as a major component of multi-subunit vaults that are ubiquitously expressed in various types of cells, is associated with the transport of miRNAs into sEVs. Under normal conditions, silencing of MVP in cytoplasm resulted in 50% reduction of RNA shuttled into exosomes but the total RNA in cells remained unchanged, while introduction of MVP increased the amount of RNA [46]. MVP knockout colon carcinoma cells led to an accumulation of cytosolic miR-193a and a decrease of miR-193a in cell-derived exosomes, while no change in miR-126a was observed [71]. These evidences suggest MVP plays a crucial role in RNA selection in sEVs. Parallel with RNA-seq data, astrocytes had higher expression of MVP than neurons. We also observed the presence of MVP in astrocytic sEVs other than neuronal sEVs. The role of MVP in shuttling RNA in sEVs released by neurons and astrocytes remains to be answered.

Annexin A2 (ANXA2) can alter the miRNAs loaded into sEVs by binding to miRNAs in a sequence-free manner [72]. Silencing ANXA2 significantly decreased the number of miRNAs loaded into sEVs but did not significantly change the miRNA profiles compared to controls, indicating ANXA2 is involved in a passive sorting. In our study, we found that the expression of ANXA2 was not significantly different between neurons and astrocytes, and neurons could incorporate more miRNAs in sEVs than astrocytes, suggesting a minimal role for ANXA2 in miRNA sorting into sEVs in neurons and astrocytes.

RBPs can bind to transcripts through common RNA elements or specific sequence motifs. Sumoylated heterogeneous nuclear ribonucleoprotein (hnRNPA2B1) can specifically bind to a short GGAG sequence on miRNAs called an EXOmotif and transfer these miRNAs into exosomes [16]. Another EXOmotif, GGCU, can be bound by synaptotogmin-binding cytoplasmic RNA-interacting protein (SYNCRIP) on miRNAs loaded into exosomes [15]. So far, more than 20 EXOmotifs have been identified for cargo loading into sEVs [73]. Therefore, we examined if the miRNAs enriched in sEVs (fold change ≥ 2 and *p*-value ≤ 0.01) secreted by neurons or astrocytes also contain such sequence motifs.

We identified two motifs in sEV-encapsulating miRNAs, CAGUAG in neuronal sEVs and GUAC in astrocytic sEVs. The GUAC motif has been reported in 7SK small nuclear RNA (7SK) as a part of the 7SK small nuclear ribonucleoprotein (7SK snRNP). This motif is required to inactivate the positive transcription elongation factor b in cells by interacting with hexamethylene bisacetamide-induced protein (HEXIM) 1, which is also incorporated in 7SK snRNP [74]. Defined synthetic single-stranded phosphorothioate oligoribonucleotides expressing the GUAC motif can strongly induce TNF-α production but not IFN-α through the binding and stimulation of the toll-like receptor 8 on human monocytes and myeloid dendritic cells but not plasmacytoid dendritic cells [75]. Both neurons and astrocytes have a similar pattern of nucleotides, CACACA, in their enriched miRNAs compared to their respective secreted sEVs. The CACACA hexamer can be efficiently bound by heterogeneous nuclear ribonucleoproteins (hnRNPs), such as hnRNP L and hnRNP LL [76–78] but this binding may require more stringent spacing between the CA repeats. Additional studies are needed to functionally validate the role of these motifs and systematically evaluate their role in sorting into neuronal and astrocytic sEVs.

## Conclusions

Our studies investigating miRNAs in sEVs secreted by primary cortical neurons and astrocytes showed donor cells selectively incorporate only a subset of cellular miRNAs into sEVs. Although the number of miRNAs detected in astrocytes were larger than those detected in neurons, neurons expressed more sEV-associated miRNAs compared to astrocytes. Distinct miRNA profiles between sEVs and their respective cells suggest the sorting of miRNAs into sEVs is precisely regulated and could be specific to cell types. Short RNA sequence motifs or EXOmotifs on the miRNAs that are differentially loaded or excluded from sEVs could have a role in being packaged into sEVs. We identified sequence motif GUAC to be enriched in astrocytic sEVs. RNA motif CACACA in miRNAs retained in neurons or astrocytes suggest a cell-type-independent mechanism to maintain cellular miRNAs. Presence of common miRNAs sorted into sEVs by both cell types suggests commonality in sorting mechanisms. Cellular RNA sequencing showed that mRNAs of five RNA-binding proteins associated with passive or active RNA sorting into sEVs were differentially expressed between neurons and astrocytes, of which MVP was highly expressed in astrocytes and their respective sEVs, while ANXA2, absent in sEVs, did not show differential expression between neurons and astrocytes. Future studies will determine the role of specific motifs and the RNA binding proteins in determining selective sorting of miRNAs into sEVs and how various stimuli or disorders can impact this packaging. Cataloging of miRNA signatures unique to CNS cells and the sEVs they release can help further investigations on the therapeutic or detrimental role of these RNA molecules in various neurological disorders. Establishing cell specific signatures, especially for neurological diseases with altered sEV-encapsulated miRNAs in biofluids, holds immense potential both as a biomarker and as a therapeutic intervention strategy.

## Supplementary Information

**Additional file 1**. Supplementary figures. **Figure S1.** Total protein level and original blots. **Figure S2.** Integrity and quality of RNA derived from neurons, astrocytes and their respective sEVs. **Figure S3.** Treemap representations of differentially expressed genes in neurons vs. astrocytes. 

**Additional file 2.** **Table S1.** miRNAs detected in neurons, astrocytes, and their respective sEVs.

**Additional file 3.** **Table S2.** Differentially expressed miRNAs in different comparison groups.

**Additional file 4.** **Table S3.** RNA sequence motifs discovered in miRNAs upregulated in each cell type and their respective sEVs.

**Additional file 5.** **Table S4.** Differentially expressed mRNAs derived from neurons and astrocytes.

## Data Availability

All data generated is included in the article and the supplementary files.

## Abbreviations

AGO1: Argonaute 1
AGO4: Argonaute 4
ANXA2: Annexin A2
BBB: Blood brain barrier
CNS: Central nervous system
DEGs: Differentially expressed genes
DMEM: Dulbecco’s modified eagle media
GFAP: Glial fibrillary acidic protein
GLAST: Glutamine aspartate transporter
GO: Gene ontology
FBS: Fetal bovine serum
FPKM: Fragments per kilobase million
HBSS: Hanks balanced salt solution
HEXIM: Hexamethylene bisacetamide-induced protein
hnRNPH1: Heterogeneous nuclear ribonucleoprotein H1
L1CAM: L1 cell adhesion molecule
MAP2: Microtubule associated protein 2
PBS: Phosphate-buffered saline
RBPs: RNA-binding proteins
sEVs: Small extracellular vesicles
snRNP: Small nuclear ribonucleoprotein
ssDNA circle: Single-stranded circular DNA
TEM: Transmission electron microscopy
TPM: Transcripts per kilobase million
ZOOPS: Zero or one site per sequence

## References

[1] van Niel G, D'Angelo G, Raposo G (2018). Shedding light on the cell biology of extracellular vesicles. Nat Rev Mol Cell Biol.

[2] Mathieu M, Martin-Jaular L, Lavieu G, Thery C (2019). Specificities of secretion and uptake of exosomes and other extracellular vesicles for cell-to-cell communication. Nat Cell Biol.

[3] Thery C, Witwer KW, Aikawa E, Alcaraz MJ, Anderson JD, Andriantsitohaina R, Antoniou A, Arab T, Archer F, Atkin-Smith GK (2018). Minimal information for studies of extracellular vesicles 2018 (MISEV2018): a position statement of the International Society for Extracellular Vesicles and update of the MISEV2014 guidelines. J Extracell Vesicles.

[4] Valadi H, Ekström K, Bossios A, Sjöstrand M, Lee JJ, Lötvall JO (2007). Exosome-mediated transfer of mRNAs and microRNAs is a novel mechanism of genetic exchange between cells. Nat Cell Biol.

[5] Bronisz A, Wang Y, Nowicki MO, Peruzzi P, Ansari K, Ogawa D, Balaj L, De Rienzo G, Mineo M, Nakano I (2014). Extracellular vesicles modulate the glioblastoma microenvironment via a tumor suppression signaling network directed by miR-1. Cancer Res.

[6] Fevrier B, Raposo G (2004). Exosomes: endosomal-derived vesicles shipping extracellular messages. Curr Opin Cell Biol.

[7] Ratajczak J, Wysoczynski M, Hayek F, Janowska-Wieczorek A, Ratajczak MZ (2006). Membrane-derived microvesicles: important and underappreciated mediators of cell-to-cell communication. Leukemia.

[8] Boukouris S, Mathivanan S (2015). Exosomes in bodily fluids are a highly stable resource of disease biomarkers. Proteom Clin Appl.

[9] Colombo M, Raposo G, Thery C (2014). Biogenesis, secretion, and intercellular interactions of exosomes and other extracellular vesicles. Annu Rev Cell Dev Biol.

[10] Hagiwara SI, Hasdemir B, Heyman MB, Chang L, Bhargava A (2019). Plasma corticotropin-releasing factor receptors and B7–2(+) extracellular vesicles in blood correlate with irritable bowel syndrome disease severity. Cells.

[11] Koppers-Lalic D, Hackenberg M, Bijnsdorp IV, van Eijndhoven MAJ, Sadek P, Sie D, Zini N, Middeldorp JM, Ylstra B, de Menezes RX (2014). Nontemplated nucleotide additions distinguish the small RNA composition in cells from exosomes. Cell Rep.

[12] Groot M, Lee H (2020). Sorting mechanisms for MicroRNAs into extracellular vesicles and their associated diseases. Cells.

[13] Tosar JP, Gambaro F, Sanguinetti J, Bonilla B, Witwer KW, Cayota A (2015). Assessment of small RNA sorting into different extracellular fractions revealed by high-throughput sequencing of breast cell lines. Nucl Acids Res.

[14] Shurtleff MJ, Temoche-Diaz MM, Karfilis KV, Ri S, Schekman R (2016). Y-box protein 1 is required to sort microRNAs into exosomes in cells and in a cell-free reaction. Elife.

[15] Santangelo L, Giurato G, Cicchini C, Montaldo C, Mancone C, Tarallo R, Battistelli C, Alonzi T, Weisz A, Tripodi M (2016). The RNA-binding protein SYNCRIP is a component of the hepatocyte exosomal machinery controlling MicroRNA sorting. Cell Rep.

[16] Villarroya-Beltri C, Gutierrez-Vazquez C, Sanchez-Cabo F, Perez-Hernandez D, Vazquez J, Martin-Cofreces N, Martinez-Herrera DJ, Pascual-Montano A, Mittelbrunn M, Sanchez-Madrid F (2013). Sumoylated hnRNPA2B1 controls the sorting of miRNAs into exosomes through binding to specific motifs. Nat Commun.

[17] Schiera G, Di Liegro CM, Di Liegro I (2015). Extracellular membrane vesicles as vehicles for brain cell-to-cell interactions in physiological as well as pathological conditions. Biomed Res Int.

[18] Ramirez SH, Andrews AM, Paul D, Pachter JS (2018). Extracellular vesicles: mediators and biomarkers of pathology along CNS barriers. Fluids Barriers CNS.

[19] Xia X, Wang Y, Huang Y, Zhang H, Lu H, Zheng JC (2019). Exosomal miRNAs in central nervous system diseases: biomarkers, pathological mediators, protective factors and therapeutic agents. Prog Neurobiol.

[20] Koopaei NN, Chowdhury EA, Jiang J, Noorani B, da Silva L, Bulut G, Hakimjavadi H, Chamala S, Bickel U, Schmittgen TD (2021). Enrichment of the erythrocyte miR-451a in brain extracellular vesicles following impairment of the blood-brain barrier. Neurosci Lett.

[21] Banks WA, Sharma P, Bullock KM, Hansen KM, Ludwig N, Whiteside TL (2020). Transport of extracellular vesicles across the blood-brain barrier: brain pharmacokinetics and effects of inflammation. Int J Mol Sci.

[22] Matsumoto J, Stewart T, Sheng L, Li N, Bullock K, Song N, Shi M, Banks WA, Zhang J (2017). Transmission of alpha-synuclein-containing erythrocyte-derived extracellular vesicles across the blood-brain barrier via adsorptive mediated transcytosis: another mechanism for initiation and progression of Parkinson's disease?. Acta Neuropathol Commun.

[23] Garcia-Romero N, Carrion-Navarro J, Esteban-Rubio S, Lazaro-Ibanez E, Peris-Celda M, Alonso MM, Guzman-De-Villoria J, Fernandez-Carballal C, de Mendivil AO, Garcia-Duque S (2017). DNA sequences within glioma-derived extracellular vesicles can cross the intact blood-brain barrier and be detected in peripheral blood of patients. Oncotarget.

[24] Fowler CD (2019). NeuroEVs: characterizing extracellular vesicles generated in the neural domain. J Neurosci.

[25] Mustapic M, Eitan E, Werner JK, Berkowitz ST, Lazaropoulos MP, Tran J, Goetzl EJ, Kapogiannis D (2017). Plasma extracellular vesicles enriched for neuronal origin: a potential window into brain pathologic processes. Front Neurosci.

[26] Goetzl EJ, Boxer A, Schwartz JB, Abner EL, Petersen RC, Miller BL, Kapogiannis D (2015). Altered lysosomal proteins in neural-derived plasma exosomes in preclinical Alzheimer disease. Neurology.

[27] Goetzl EJ, Mustapic M, Kapogiannis D, Eitan E, Lobach IV, Goetzl L, Schwartz JB, Miller BL (2016). Cargo proteins of plasma astrocyte-derived exosomes in Alzheimer's disease. FASEB J.

[28] Winston CN, Romero HK, Ellisman M, Nauss S, Julovich DA, Conger T, Hall JR, Campana W, O’Bryant SE, Nievergelt CM (2019). Assessing neuronal and astrocyte derived exosomes from individuals with mild traumatic brain injury for markers of neurodegeneration and cytotoxic activity. Front Neurosci.

[29] Picciolini S, Gualerzi A, Vanna R, Sguassero A, Gramatica F, Bedoni M, Masserini M, Morasso C (2018). Detection and characterization of different brain-derived subpopulations of plasma exosomes by surface plasmon resonance imaging. Anal Chem.

[30] Honegger A, Schilling D, Bastian S, Sponagel J, Kuryshev V, Sultmann H, Scheffner M, Hoppe-Seyler K, Hoppe-Seyler F (2015). Dependence of intracellular and exosomal microRNAs on viral E6/E7 oncogene expression in HPV-positive tumor cells. PLoS Pathog.

[31] Huang Q, Yang J, Zheng J, Hsueh C, Guo Y, Zhou L (2018). Characterization of selective exosomal microRNA expression profile derived from laryngeal squamous cell carcinoma detected by next generation sequencing. Oncol Rep.

[32] McDonald MK, Tian Y, Qureshi RA, Gormley M, Ertel A, Gao R, Aradillas Lopez E, Alexander GM, Sacan A, Fortina P, Ajit SK (2014). Functional significance of macrophage-derived exosomes in inflammation and pain. Pain.

[33] McDonald MK, Capasso KE, Ajit SK (2013). Purification and microRNA profiling of exosomes derived from blood and culture media. J Vis Exp.

[34] Thery C, Amigorena S, Raposo G, Clayton A. Isolation and characterization of exosomes from cell culture supernatants and biological fluids. Curr Protoc Cell Biol. 2006; Chapter 3:Unit 3 22. 10.1002/0471143030.cb0322s30 18228490

[35] Zhu FY, Chen MX, Ye NH, Qiao WM, Gao B, Law WK, Tian Y, Zhang D, Zhang D, Liu TY (2018). Comparative performance of the BGISEQ-500 and Illumina HiSeq4000 sequencing platforms for transcriptome analysis in plants. Plant Methods.

[36] Fehlmann T, Reinheimer S, Geng C, Su X, Drmanac S, Alexeev A, Zhang C, Backes C, Ludwig N, Hart M (2016). cPAS-based sequencing on the BGISEQ-500 to explore small non-coding RNAs. Clin Epigenet.

[37] Nawrocki EP, Eddy SR (2013). Infernal 1.1: 100-fold faster RNA homology searches. Bioinformatics.

[38] Love MI, Huber W, Anders S (2014). Moderated estimation of fold change and dispersion for RNA-seq data with DESeq2. Genome Biol.

[39] da Huang W, Sherman BT, Lempicki RA (2009). Systematic and integrative analysis of large gene lists using DAVID bioinformatics resources. Nat Protoc.

[40] da Huang W, Sherman BT, Lempicki RA (2009). Bioinformatics enrichment tools: paths toward the comprehensive functional analysis of large gene lists. Nucl Acids Res.

[41] Supek F, Bosnjak M, Skunca N, Smuc T (2011). REVIGO summarizes and visualizes long lists of gene ontology terms. PLoS ONE.

[42] Everaert C, Luypaert M, Maag JLV, Cheng QX, Dinger ME, Hellemans J, Mestdagh P (2017). Benchmarking of RNA-sequencing analysis workflows using whole-transcriptome RT-qPCR expression data. Sci Rep.

[43] Bailey TL, Boden M, Buske FA, Frith M, Grant CE, Clementi L, Ren J, Li WW, Noble WS (2009). MEME SUITE: tools for motif discovery and searching. Nucl Acids Res.

[44] Lewis BP, Shih IH, Jones-Rhoades MW, Bartel DP, Burge CB (2003). Prediction of mammalian microRNA targets. Cell.

[45] Crescitelli R, Lasser C, Szabo TG, Kittel A, Eldh M, Dianzani I, Buzas EI, Lotvall J. Distinct RNA profiles in subpopulations of extracellular vesicles: apoptotic bodies, microvesicles and exosomes. J Extracell Vesicles 2013, 2. 10.3402/jev.v2i0.20677 PMC3823106 24223256

[46] Statello L, Maugeri M, Garre E, Nawaz M, Wahlgren J, Papadimitriou A, Lundqvist C, Lindfors L, Collen A, Sunnerhagen P (2018). Identification of RNA-binding proteins in exosomes capable of interacting with different types of RNA: RBP-facilitated transport of RNAs into exosomes. PLoS ONE.

[47] Upadhya R, Zingg W, Shetty S, Shetty AK (2020). Astrocyte-derived extracellular vesicles: neuroreparative properties and role in the pathogenesis of neurodegenerative disorders. J Control Release.

[48] Sweeney MD, Sagare AP, Zlokovic BV (2018). Blood–brain barrier breakdown in Alzheimer disease and other neurodegenerative disorders. Nat Rev Neurol.

[49] Gomez-Molina C, Sandoval M, Henzi R, Ramirez JP, Varas-Godoy M, Luarte A, Lafourcade CA, Lopez-Verrilli A, Smalla KH, Kaehne T, Wyneken U (2019). Small extracellular vesicles in rat serum contain astrocyte-derived protein biomarkers of repetitive stress. Int J Neuropsychopharmacol.

[50] Zhang Y, Kim MS, Jia B, Yan J, Zuniga-Hertz JP, Han C, Cai D (2017). Hypothalamic stem cells control ageing speed partly through exosomal miRNAs. Nature.

[51] Neckles VN, Morton MC, Holmberg JC, Sokolov AM, Nottoli T, Liu D, Feliciano DM (2019). A transgenic inducible GFP extracellular-vesicle reporter (TIGER) mouse illuminates neonatal cortical astrocytes as a source of immunomodulatory extracellular vesicles. Sci Rep.

[52] Chaudhuri AD, Dastgheyb RM, Yoo S-W, Trout A, Talbot CC, Hao H, Witwer KW, Haughey NJ (2018). TNFα and IL-1β modify the miRNA cargo of astrocyte shed extracellular vesicles to regulate neurotrophic signaling in neurons. Cell Death Dis.

[53] Jovicic A, Gitler AD (2017). Distinct repertoires of microRNAs present in mouse astrocytes compared to astrocyte-secreted exosomes. PLoS ONE.

[54] Jovicic A, Roshan R, Moisoi N, Pradervand S, Moser R, Pillai B, Luthi-Carter R (2013). Comprehensive expression analyses of neural cell-type-specific miRNAs identify new determinants of the specification and maintenance of neuronal phenotypes. J Neurosci.

[55] Mannironi C, Biundo A, Rajendran S, De Vito F, Saba L, Caioli S, Zona C, Ciotti T, Caristi S, Perlas E (2018). miR-135a regulates synaptic transmission and anxiety-like behavior in amygdala. Mol Neurobiol.

[56] Zhang ZB, Tan YX, Zhao Q, Xiong LL, Liu J, Xu FF, Xu Y, Bobrovskaya L, Zhou XF, Wang TH (2019). miRNA-7a-2-3p inhibits neuronal apoptosis in oxygen-glucose deprivation (OGD) model. Front Neurosci.

[57] Li Y, Zhao Y, Cheng M, Qiao Y, Wang Y, Xiong W, Yue W (2018). Suppression of microRNA-144–3p attenuates oxygen-glucose deprivation/reoxygenation-induced neuronal injury by promoting Brg1/Nrf2/ARE signaling. J Biochem Mol Toxicol.

[58] Sakai A, Saitow F, Miyake N, Miyake K, Shimada T, Suzuki H (2013). miR-7a alleviates the maintenance of neuropathic pain through regulation of neuronal excitability. Brain.

[59] Varol D, Mildner A, Blank T, Shemer A, Barashi N, Yona S, David E, Boura-Halfon S, Segal-Hayoun Y, Chappell-Maor L (2017). Dicer deficiency differentially impacts microglia of the developing and adult brain. Immunity.

[60] Zhang M, Shi Y, Zhang Y, Wang Y, Alotaibi F, Qiu L, Wang H, Peng S, Liu Y, Li Q (2020). miRNA-5119 regulates immune checkpoints in dendritic cells to enhance breast cancer immunotherapy. Cancer Immunol Immunother.

[61] Guo D, Ma J, Li T, Yan L (2018). Up-regulation of miR-122 protects against neuronal cell death in ischemic stroke through the heat shock protein 70-dependent NF-kappaB pathway by targeting FOXO3. Exp Cell Res.

[62] Lukiw WJ (2012). Evolution and complexity of micro RNA in the human brain. Front Genet.

[63] Temoche-Diaz MM, Shurtleff MJ, Nottingham RM, Yao J, Fadadu RP, Lambowitz AM, Schekman R (2019). Distinct mechanisms of microRNA sorting into cancer cell-derived extracellular vesicle subtypes. Elife.

[64] Mukherjee K, Ghoshal B, Ghosh S, Chakrabarty Y, Shwetha S, Das S, Bhattacharyya SN (2016). Reversible HuR-microRNA binding controls extracellular export of miR-122 and augments stress response. EMBO Rep.

[65] Shams R, Saberi S, Zali M, Sadeghi A, Ghafouri-Fard S, Aghdaei HA (2020). Identification of potential microRNA panels for pancreatic cancer diagnosis using microarray datasets and bioinformatics methods. Sci Rep.

[66] Zeng L, Wu JL, Liu LM, Jiang JQ, Wu HJ, Zhao M, Lu QJ (2018). Serum miRNA-371b-5p and miRNA-5100 act as biomarkers for systemic lupus erythematosus. Clin Immunol.

[67] Smirnova L, Grafe A, Seiler A, Schumacher S, Nitsch R, Wulczyn FG (2005). Regulation of miRNA expression during neural cell specification. Eur J Neurosci.

[68] Jean-Toussaint R, Lin Z, Tian Y, Gupta R, Pande R, Luo X, Hu H, Sacan A, Ajit SK (2021). Therapeutic and prophylactic effects of macrophage-derived small extracellular vesicles in the attenuation of inflammatory pain. Brain Behav Immun.

[69] You Y, Borgmann K, Edara VV, Stacy S, Ghorpade A, Ikezu T (2020). Activated human astrocyte-derived extracellular vesicles modulate neuronal uptake, differentiation and firing. J Extracell Vesicles.

[70] Sharma K, Schmitt S, Bergner CG, Tyanova S, Kannaiyan N, Manrique-Hoyos N, Kongi K, Cantuti L, Hanisch U-K, Philips M-A (2015). Cell type– and brain region–resolved mouse brain proteome. Nat Neurosci.

[71] Teng Y, Ren Y, Hu X, Mu J, Samykutty A, Zhuang X, Deng Z, Kumar A, Zhang L, Merchant ML (2017). MVP-mediated exosomal sorting of miR-193a promotes colon cancer progression. Nat Commun.

[72] Hagiwara K, Katsuda T, Gailhouste L, Kosaka N, Ochiya T (2015). Commitment of annexin A2 in recruitment of microRNAs into extracellular vesicles. FEBS Lett.

[73] Basso M, Bonetto V (2016). Extracellular vesicles and a novel form of communication in the brain. Front Neurosci.

[74] Fujinaga K, Luo Z, Peterlin BM (2014). Genetic analysis of the structure and function of 7SK small nuclear ribonucleoprotein (snRNP) in cells. J Biol Chem.

[75] Forsbach A, Nemorin JG, Montino C, Muller C, Samulowitz U, Vicari AP, Jurk M, Mutwiri GK, Krieg AM, Lipford GB, Vollmer J (2008). Identification of RNA sequence motifs stimulating sequence-specific TLR8-dependent immune responses. J Immunol.

[76] Smith SA, Ray D, Cook KB, Mallory MJ, Hughes TR, Lynch KW (2013). Paralogs hnRNP L and hnRNP LL exhibit overlapping but distinct RNA binding constraints. PLoS ONE.

[77] Zhang W, Zeng F, Liu Y, Zhao Y, Lv H, Niu L, Teng M, Li X (2013). Crystal structures and RNA-binding properties of the RNA recognition motifs of heterogeneous nuclear ribonucleoprotein L: insights into its roles in alternative splicing regulation. J Biol Chem.

[78] Rahman MA, Masuda A, Ohe K, Ito M, Hutchinson DO, Mayeda A, Engel AG, Ohno K (2013). HnRNP L and hnRNP LL antagonistically modulate PTB-mediated splicing suppression of CHRNA1 pre-mRNA. Sci Rep.

