# Supplementary material for: Differential RNA packaging into small extracellular vesicles by neurons and astrocytes
Source: Cell Commun Signal. 2021 Jul 10;19:75. doi: 10.1186/s12964-021-00757-4 (PMC8272329; doi:10.1186/s12964-021-00757-4)
Supplement: Supplementary file 2 — Additional file 1. Supplementary figures. Figure S1. Total protein level and original blots. Figure S2. Integrity and quality of RNA derived from neurons, astrocytes and their respective sEVs. Figure S3. Treemap representations of differentially expressed genes in neurons vs. astrocytes. [file 12964_2021_757_MOESM2_ESM.docx]

**Supplementary Figures**

**Supplementary Figure 1**

**
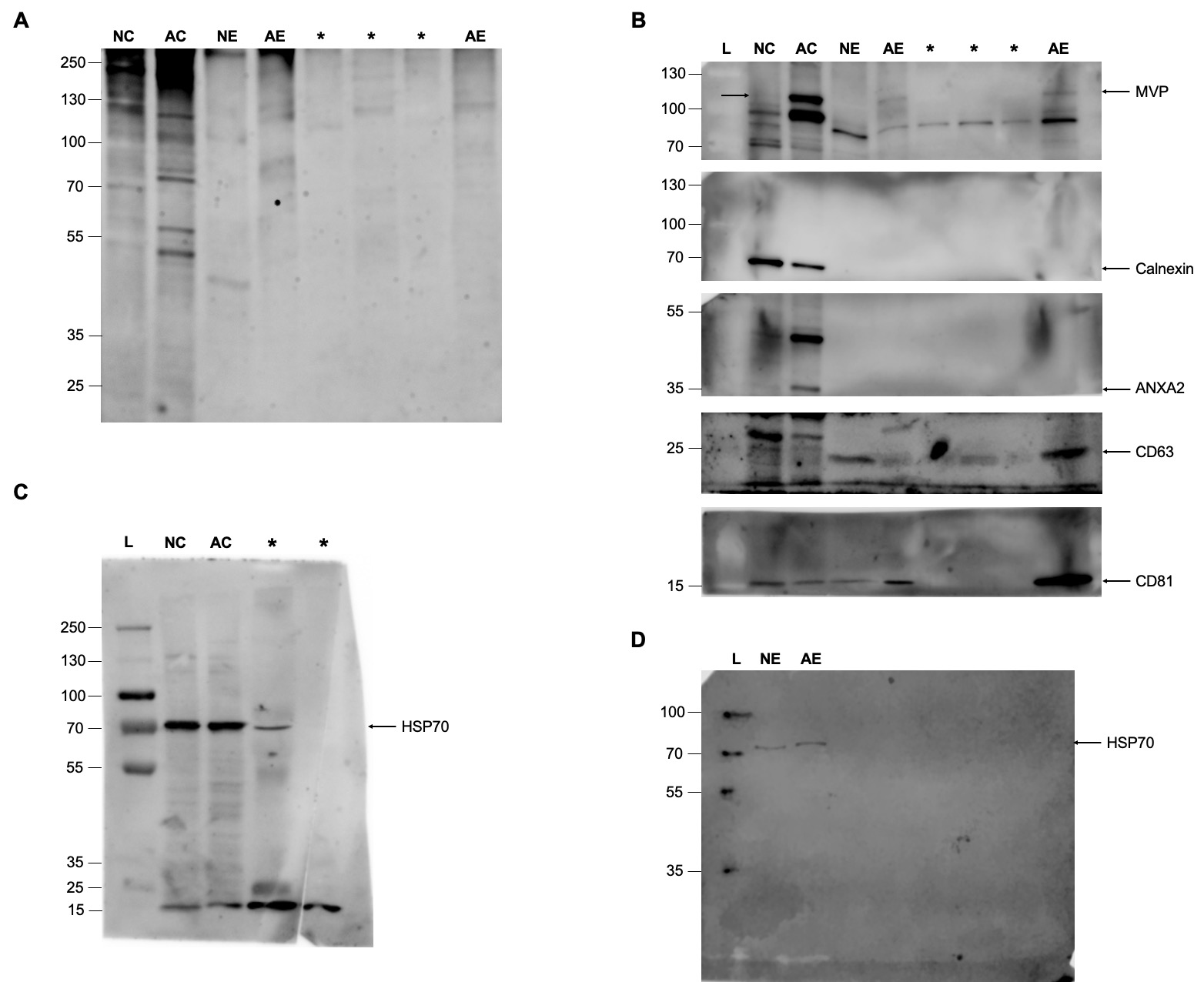
**

**Supplementary Figure 1 Total protein level and original blots. (A)** Total protein loading of neurons, astrocytes, and their sEVs shown by gel staining. Ten μg of total protein were loaded on to the gel. **(B)** Original blot of calnexin, CD63, and CD81 (shown in Figure 2D), as well as MVP and ANXA2 (shown in Figure 6C). Original blots of HSP70 from neurons and astrocytes **(C)** as well as their sEVs **(D)** shown in Figure 2D. Arrows indicate the target proteins. Asterisks represent samples tested for another study. L: molecular weight protein ladder, NC: neurons, AC: astrocytes, NE: sEVs from neurons, AE: sEVs from astrocytes, MVP: major vault protein, ANXA2: annexin A2.

**Supplementary Figure 2**


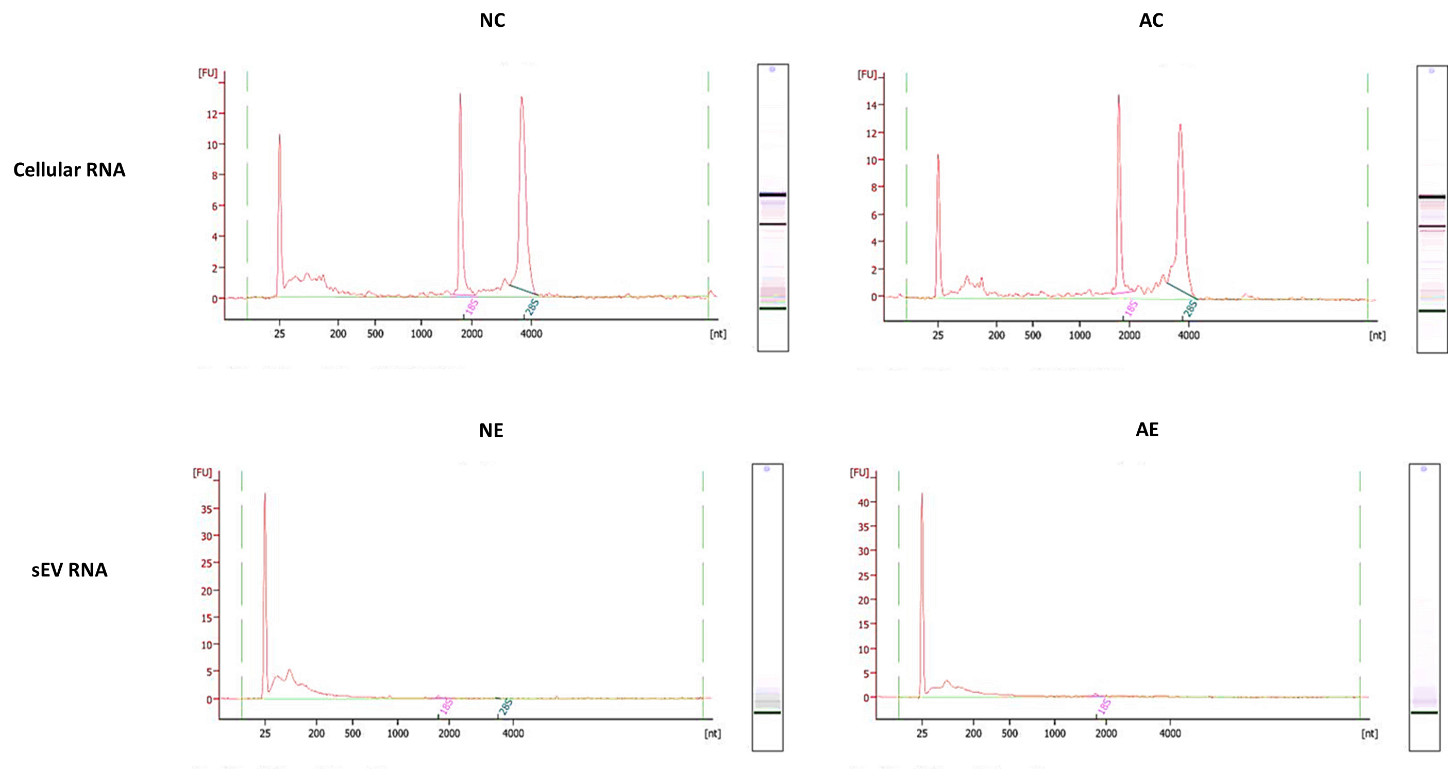


**Supplementary Figure 2** **Integrity and quality of RNA derived from neurons, astrocytes and their respective sEVs.** Cellular RNA for both neurons and astrocytes had prominent 18S and 28S peaks but absent in corresponding sEVs. The sEV samples had peaks around 25 – 200 nucleotides, indicating the presence of small RNAs. NC: neurons, AC: astrocytes, NE: sEVs from neurons, AE: sEVs from astrocytes.

**Supplementary Figure 3**

**
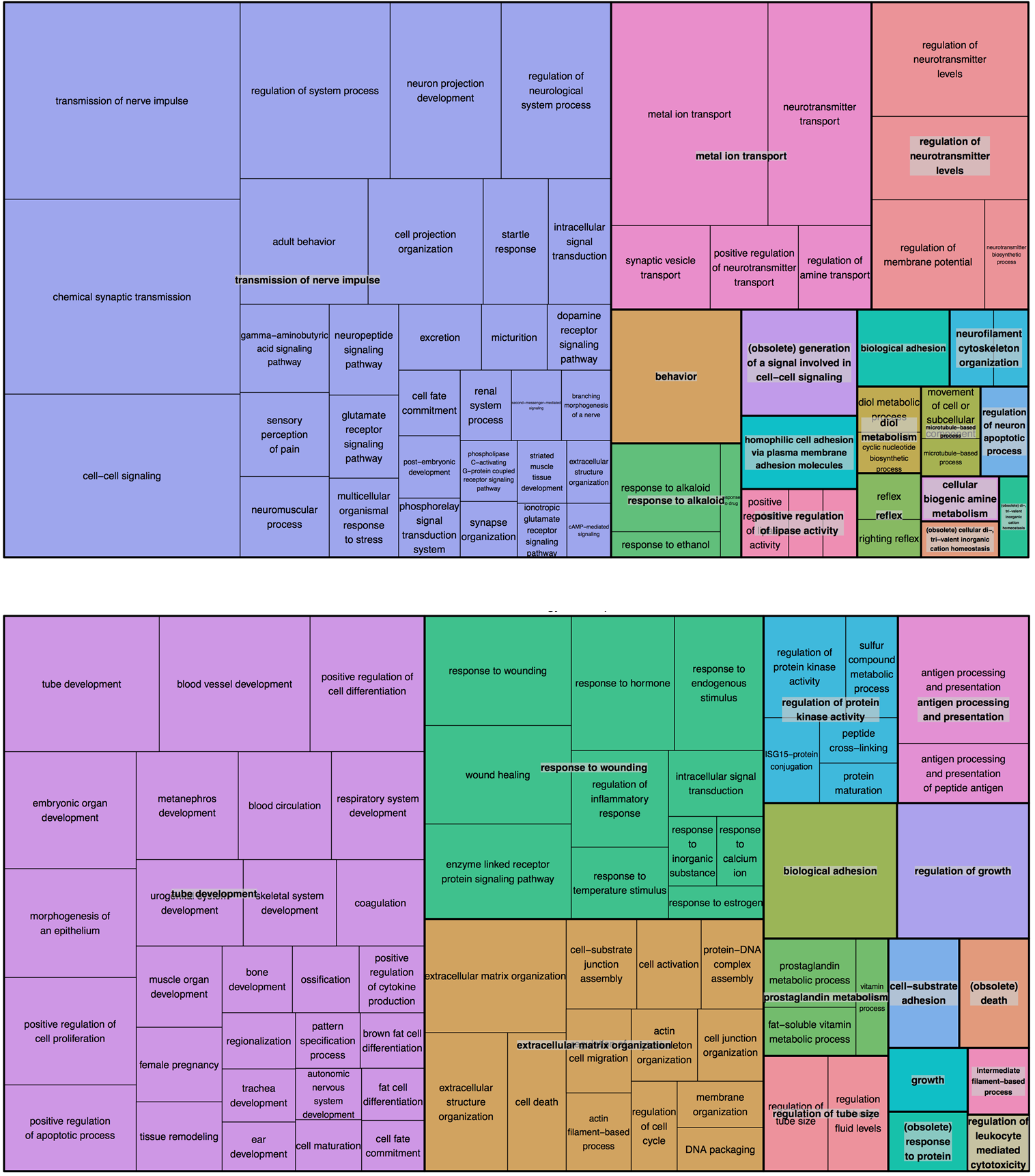
**

**Supplementary Figure 3 Treemap representations of differentially expressed genes in neurons vs. astrocytes.** **(Upper)** Biological processes enriched for mRNAs that are upregulated in neurons. **(Lower)** Biological processes enriched for mRNAs upregulated in astrocytes.
